# System of Diseases of the Eye

**Published:** 1899-09

**Authors:** 


**System of Diseases of the Eye.** Edited by WILLIAM F. NORRIS, M.D., and CHARLES A. OLIVER, M.D. Vol. III.—*Local Diseases, Glaucoma, Wounds and Injuries, Operations.* Pp. xii., 962. Philadelphia: J. B. Lippincott Company. [1898.]

A considerable departure has been made from the original forecast of the contents of this volume. The articles on diseases of the cornea, diseases of the lens, and errors of refraction have been deferred to the fourth volume. On the other hand, Dr. G. C. Harlan, in addition to his chapter on "Diseases of the Eyelids," contributes forty-four pages on "Operations performed upon the Eyelids," a useful section, which was not specified in the original programme, and which adequately describes all the best-known expedients. For want of space a critical reference to the various articles is impossible, but some 160 pages at the end of the volume on "operations usually performed in eye-surgery," deserve special mention. The author, Dr. Herman Knapp, is so well known as a master of this subject that his dogmatic statements are more than justified, though all may not agree with them. For example, to cleanse the eye before operation he uses bichloride of mercury, 1 to 5000, with which he mops the everted palpebral conjunctiva, while in cataract operation he advocates operating on the patient in his bed. His style is terse; but the meaning is precise, and the article covers an immense amount of ground. Recognising the risks of a needling operation, but at the same time its almost inevitable necessity, he performs his extractions specially with that end in view, and takes particular care to avoid any unnecessary mangling of the iris, or injury to the lens capsule. The disastrous suppuration which is occasionally seen to follow discission of secondary cataract he attributes not to direct inoculation from the needle, or immediate infection of the wound, but to the awakening of pyogenic germs which had been previously introduced, and were lying dormant.

The volume on the whole is remarkably free from errors. We notice, however, on page 261, in speaking of pupillometers, a correct description is given of the Keratometer, invented by Mr. Priestley Smith some fifteen years ago, which the writer says may be used as a pupillometer. But by accident it is called a Tonometer, and as a result a picture of the tonometer, invented by Priestley Smith in 1866, is dragged in "to render the working of the tonometer clear to readers." It is incredible that the authors of the article should be responsible for the whole of this blunder. The chapter on glaucoma by Priestley Smith, in which the tonometer is again depicted and correctly described, is admirable in every way, and the wood-cut illustrations are all that could be wished.

In reference to the illustrations of the volume generally, it must be said that they are on the whole disappointing. Some are very good. Some are pretentious, but convey no particular information; whilst others, particularly those illustrating ophthalmoscopic appearances, are quite unworthy of being included in so important a work. The article on diseases of the retina is marred by such plates as XII. and XXVII. The section on diseases of the choroid has no illustrations of the fundus oculi, and so does not suffer in this respect, but it is only fair to say that the illustration of the fundus facing page 768 leaves nothing to be desired.

